# How is theory of mind useful? Perhaps to enable social pretend play

**DOI:** 10.3389/fpsyg.2015.01559

**Published:** 2015-10-15

**Authors:** Rebecca A. Dore, Eric D. Smith, Angeline S. Lillard

**Affiliations:** ^1^Department of Psychology, University of VirginiaCharlottesville, VA, USA; ^2^Department of Psychology, Murray State UniversityMurray, KY, USA

**Keywords:** theory of mind, pretend play, play ethos, mental states, simulation theory

It is often claimed that theory of mind (ToM) is facilitated by pretend play (PP), or by a particular type of PP, social pretend play (SPP). Here we challenge that view, proposing instead that ToM might be useful for driving SPP, rather than the reverse. We discuss background theory, review pertinent studies, and explain why the “ToM first” view is at least equally likely.

The first form of pretend to consider is solitary PP, emerging at 12–18 months, in which one engages in three basic types of transformation: substitutes one object for another, projects imaginary characteristics onto objects, and imagines situations that do not exist (Leslie, 1987). The second form is social pretend play (SPP), which involves the same basic transformations but occurs with others, and emerges around age 3. We address these two forms of PP consecutively.

There is a fundamental similarity, or isomorphism (Leslie, 1987), between PP and ToM. For all three types of transformation just described, PP involves projecting a different reality onto a situation, all while knowing what the real situation is (Lillard, 1993). For example, a child might mentally project a telephone onto a banana, engaging in both of the first two types of transformation. Similarly, understanding false belief—a foundational skill in ToM—involves understanding that someone is projecting a different reality onto a situation, when one knows what the real situation is. For example, a child might see someone as mentally projecting a chocolate bar inside a drawer, while the chocolate bar is actually in the cupboard.

Theoretically, then, the ability to imagine a situation that differs from one's present reality underlies both PP and understanding others' minds, in particular, false belief (Leslie, 1987; Flavell, 1988; Forguson and Gopnik, 1988; Moses and Chandler, 1992). Leslie (1987) argued that the cognitive architecture that enables one to engage in PP also enables one to understand false belief, suggesting that PP should be related to ToM.

Hence it is possible that PP engenders ToM by giving children practice at projecting representations that differ from reality onto that reality. In pursuit of evidence on this point, many researchers have examined the correlation between PP and ToM. For example, Taylor and Carlson (1997) assessed preschoolers' PP sophistication by examining their tendency to use imaginary objects in pretend action sequences (e.g., holding an imaginary toothbrush to brush one's teeth rather than using a finger as a toothbrush). The researchers examined whether this more sophisticated form of PP correlated with children's performance on standard ToM assessments. However, across many studies, findings are inconsistent: Although some observe significant relationships (Suddendorf et al., 1999; Nielsen and Dissanayake, 2000) others do not, even with the same measures and very similar samples (Schwebel et al., 1999; Lillard, 2001b). Even within studies that found that solitary PP and ToM were related, inconsistency was observed among measures. For example, Taylor and Carlson (1997) found that ToM correlated with self- but not object-directed pretense, and Lalonde and Chandler (1995) found a significant relationship for “engages in simple make-belief actions alone” but not for “uses … objects for make-believe activities alone” (p. 395). Cumulatively, these inconsistent findings undermine the idea of a reliable causal relationship between solitary PP and ToM.

This brings us to the second type of PP, social PP. When children engage in SPP, they pretend to be other people with distinct thoughts and emotions. A major theory of how we understand others' minds, simulation theory, suggests that pretending to be someone else aids ToM because it gives children practice in simulating others' mental states and imagining oneself in another's shoes (Harris, 1995). If pretending to be others does involve such metarepresentations, it would be expected to predict ToM (Lillard, 2001a).

Indeed, researchers often find correlations between SPP and ToM. For example, Astington and Jenkins (1995) assessed the sophistication of preschoolers' pretend play through coding their explicit role assignments (e.g., “I'll be the mommy”) and joint proposals (e.g., “Let's go to the store”). These behaviors were associated with children's ToM. Several earlier studies are also consistent with these findings (Rubin and Maioni, 1975; Connolly and Doyle, 1984; Peisach and Hardeman, 1985; Lalonde and Chandler, 1995). Another type of SPP is having an imaginary companion. When children postulate imagined beings, they must represent those beings' mental states. Indeed, Taylor and Carlson (1997) found correlations between having an imaginary companion and ToM (see also Giménez-Dasí et al., 2014).

However, just as for PP, results are inconsistent (Cole and LaVoie, 1985). Some of the same studies that find positive relationships for certain ToM measures report null results for other measures. For example, in the studies cited above, PP correlated with spatial but not affective perspective taking (Rubin and Maioni, 1975), affective but not cognitive perspective taking (Connolly and Doyle, 1984), and to appearance reality but not false belief (Schwebel et al., 1999). Other research revealed inconsistent relationships between ToM and different pretense measures. For example, although Youngblade and Dunn (1995) found that ToM was related to role enactment, it was not related to explicit role assignments, in contrast to Astington and Jenkins (1995), who found that ToM was related to joint proposals and role assignments but not to a more general measure of social pretense. At the very least, such inconsistent relationships suggest that SPP does not reliably improve ToM.

Nonetheless, a single-time-point correlational study is not an ideal design for inferring causality. Lagged designs, although imperfect, are better, and some longitudinal studies have found evidence for a causal relationship. One found that children's SPP at 33 months predicted ToM at 40 months (Youngblade and Dunn, 1995). Another with four time points and several different measures of PP and SPP found that certain aspects of PP related to concurrent ToM and predicted ToM over time (Lillard and Kavanaugh, 2014). However, not all measures were related at all time points. For example, pretense production at 24 months (in a social situation with the mother) predicted ToM at 48 months but not at 60 months, and sophistication of the child's play narrative with an experimenter at 36 months predicted (at trend-level) ToM at 60 but not 48 months.

However, neither of these studies investigated whether these lagged relationships could be explained differently, namely, whether earlier ToM might foster both SPP earlier, and ToM later. In this case, it might look as if SPP at Time 1 leads to ToM at Time 2, when in fact SPP at Time 1 was simply a by-product of having more advanced ToM at Time 1. We know of only one study that could shed light on this, by also testing ToM at Time 1 (Jenkins and Astington, 2000). In this study earlier ToM predicted later SPP, whereas earlier SPP did not predict later ToM, suggesting that better ToM might enable children to engage in more, and more advanced, PP.

Although these findings are suggestive, the most solid evidence for a causal effect of SPP on ToM would come from training studies, in which some children experience an intervention to promote SPP and then are compared to an otherwise matched control group who did not. Indeed, several such studies have reported that SPP training improves ToM or related skills (Rosen, 1974; Saltz and Johnson, 1974; Fink, 1976; Saltz et al., 1977; Burns and Brainerd, 1979; Dockett, 1998). However, these studies all had significant methodological shortcomings (see Lillard et al., 2013), including unmasked experimenters who may have influenced posttest performance and unequal adult contact between groups. A study designed to rule out these possible confounds found no improvement in ToM for children in a SPP training group (Smith et al., 1981).

In a recent study, Qu et al. (2015) reported that SPP improves ToM, but given the training content, this conclusion is questionable. In four, 45 min sessions, kindergartners were read books with distinctly ToM-related scenarios (e.g., a child has a false belief about where her books are) and then acted out the stories. Controlling for pretest ToM skills, children who had this experience had better posttest ToM compared to children in a free play control group. Although the particular training experience described here appears to be effective in improving ToM, the conclusion that, in general, children's naturalistic SPP leads to better ToM goes beyond the data.

Furthermore, we recently failed to show evidence for the proposed simulation mechanism (Dore and Lillard, unpublished raw data, 2013). We reasoned that if SPP helps ToM by causing children to think about mental states, then SPP should prime mental states, leading to a stronger orientation toward mental states afterwards. In two studies, children played with an experimenter for 10 min. In a doll condition, the experimenter guided children through a partially scripted play session focusing on role play, cognition, and emotions with Playmobil dolls and animals. In a block condition with a Magneato construction set, the experimenter avoided using mental state language and directed play away from any child-generated pretense. Before and after the play session, children were given a task designed to assess their preference for thinking about people in terms of mental states vs. behaviors (Lillard and Flavell, 1990). Children in the doll condition did not describe people in terms of their mental states any more than did children in the block condition. Although null results should be interpreted with caution, this data from two studies we conducted (*N* = 100) failed to support a mechanism by which SPP might improve ToM: by causing children to focus on mental states.

Overall, the evidence described here is not strongly supportive of a causal relationship in which PP promotes ToM skills. (For a more detailed discussion of studies up to 2013, we urge readers to see the Theory of Mind section of Lillard et al., 2013.) Two other possibilities should be given equal consideration: a third variable underpinning both ToM and SPP, and reverse directionality, in which better ToM skills enable SPP—making SPP one reason why ToM is useful. One longitudinal study finds this reverse relationship (Jenkins and Astington, 2000), as described above, and more studies should be designed to test this possibility.

Given the state of the research, why is it so often assumed that PP helps ToM (Leslie, 1987; Flavell, 1988; Forguson and Gopnik, 1988; Moses and Chandler, 1992; Harris, 2000; Lillard, 2001a; Bergen, 2002; Ashiabi, 2007; Wellman, 2014)? Researchers might be swayed by what Smith (1988) called “play ethos,” the strong belief that PP promotes development. When researchers design, run, and interpret their empirical investigations from the perspective that PP must help development, it seems highly possible that their beliefs might unintentionally bias their findings. We note that researchers who have found significant correlations between PP and ToM tend to frame their findings in the context of a positive effect of PP and the training studies described here are clearly designed with this causal direction in mind. Training studies designed to assess the reverse causal relationship might train ToM and examine whether PP, or sophistication of PP, increases. Longitudinal studies in natural settings could be designed to be able to assess both directions of causality, controlling for potentially related variables like verbal ability. Finally, future training studies designed to assess the potential causal role of PP in ToM development can take care to avoid the methodological problems common in this literature.

Why is the relationship between PP and ToM an important one to pin down? Surely, even confidence that the causal view is incorrect should not lead one to conclude that PP does not have value in children's lives in other ways, either to promote well-being (Lillard et al., 2013), for relaxation (Hutt et al., 1989), to promote positive adult-child interaction (Paley, 2005) or even for fun (Power, 2000). However, embracing the idea that PP promotes ToM without solid evidence is not beneficial, and may even be indirectly harmful if such efforts detract from undertakings with better-established effectiveness. More and better research to clarify the relationship between these complex activities will have important theoretical and practical applications.

## Conflict of interest statement

The authors declare that the research was conducted in the absence of any commercial or financial relationships that could be construed as a potential conflict of interest.

## References

[B1] Ashiabi G. S. (2007). Play in the preschool classroom: its socioemotional significance and the teacher's role in play. Early Childhood Educ. J. 35, 199–207. 10.1007/s10643-007-0165-8

[B2] Astington J. W. Jenkins J. M. (1995). Theory of mind development and social understanding. Cogn. Emot. 9, 151–165. 10.1080/02699939508409006

[B3] Bergen D. (2002). The role of pretend play in children's cognitive development. Early Childhood 4, 1–13.

[B4] Burns S. M. Brainerd C. J. (1979). Effects of constructive and dramatic play on perspective-taking in very young children. Dev. Psychol. 15, 512–521. 10.1037/0012-1649.15.5.512

[B5] Cole D. LaVoie J. C. (1985). Fantasy play and related cognitive development in 2- to 6-year-olds. Dev. Psychol. 21, 233–240. 10.1037/0012-1649.21.2.233

[B6] Connolly J. A. Doyle A.-B. (1984). Relation of social fantasy play to social competence in preschoolers. Dev. Psychol. 20, 797–806. 10.1037/0012-1649.20.5.797

[B7] Dockett S. (1998). Constructing understandings through play in the early years. Int. J. Early Years Educ. 6, 105–116. 10.1080/0966976980060109

[B8] Fink R. S. (1976). Role of imaginative play in cognitive development. Psychol. Rep. 39, 895–906. 10.2466/pr0.1976.39.3.895

[B9] Flavell J. H. (1988). The development of children's knowledge about the mind: from cognitive connections to mental representations, in Developing Theories of Mind, eds Astington J. W. Harris P. L. Olson D. R. (Cambridge: Cambridge University Press), 244–267.

[B10] Forguson L. Gopnik A. (1988). The ontogeny of common sense, in Developing Theories of Mind, eds Astington J. W. Harris P. L. Olson D. R. (New York, NY: Cambridge University Press), 226–243.

[B11] Giménez-Dasí M. Pons F. Bender P. K. (2014). Imaginary companions, theory of mind and emotion understanding in young children. Europ. Early Childhood Educ. Res. J. 10.1080/1350293X.2014.919778 [Epub ahead of print].

[B12] Harris P. L. (1995). From simulation to folk psychology: the case for development, in Folk psychology, Vol. 3, eds Davies M. Stone T. (Cambridge: Blackwell), 207–221.

[B13] Harris P. L. (2000). The Work of the Imagination. Oxford: Blackwell.

[B14] Hutt S. J. Tyler S. Hutt C. Christopherson H. (1989). Play, Exploration, and Learning: A Natural History of the Preschool. London: Routledge.

[B15] Jenkins J. M. Astington J. W. (2000). Theory of mind and social behavior: causal models tested in a longitudinal study. Merrill Palmer Q. 46, 203–220.

[B16] Lalonde C. Chandler M. (1995). False belief understanding goes to school: on the social-emotional consequences of coming early or late to a first theory of mind. Cogn. Emot. 9, 167–185. 10.1080/0269993950840900726394990

[B17] Leslie A. M. (1987). Pretense and representation: the origins of “theory of mind”. Psychol. Rev. 94, 412–426. 10.1037/0033-295X.94.4.412

[B18] Lillard A. S. (1993). Pretend play skills and the child's theory of mind. Child Dev. 64, 348–371. 10.2307/1131255 8477622

[B19] Lillard A. S. (2001a). Pretend play as twin earth. Dev. Rev. 21, 1–33. 10.1006/drev.2001.0532

[B20] Lillard A. S. (2001b). Pretending, understanding pretense, and understanding minds, in Play and Culture Studies, Vol. 3, ed Reifel S. (Westport, CT: Ablex), 233–254.

[B21] Lillard A. S. Flavell J. H. (1990). Young children's preference for mental state versus behavioral descriptions of human action. Child Dev. 61, 731–741. 10.1111/j.1467-8624.1990.tb02816.x 2364748

[B22] Lillard A. S. Kavanaugh R. D. (2014). The contribution of symbolic skills to the development of an explicit theory of mind. Child Dev. 85, 1535–1551. 10.1111/cdev.12227 24502297

[B23] Lillard A. S. Lerner M. D. Hopkins E. J. Dore R. A. Smith E. D. Palmquist C. M. (2013). The impact of pretend play on children's development: a review of the evidence. Psychol. Bull. 139, 1–34. 10.1037/a0029321 22905949

[B24] Moses L. Chandler M. (1992). Traveler's guide to children's theories of mind. Psychol. Inq. 3, 286–301. 10.1207/s15327965pli0303_22

[B25] Nielsen M. Dissanayake C. (2000). An investigation of pretend play, mental state terms and false belief understanding: in search of a metarepresentational link. Br. J. Dev. Psychol. 18, 609–624. 10.1348/026151000165887

[B26] Paley V. G. (2005). A Child's Work: The Importance of Fantasy Play. Chicago, IL: University of Chicago Press.

[B27] Peisach E. Hardeman M. (1985). Imaginative play and logical thinking in young children. J. Genet. Psychol. 146, 233–248. 10.1080/00221325.1985.9914451

[B28] Power T. G. (2000). Play and Exploration in Children and Animals. Mahwah, NJ: Erlbaum.

[B29] Qu L. Shen P. Chee Y. Y. Chen L. (2015). Teachers' theory-of-mind coaching and children's executive function predict the training effect of sociodramatic play on children's theory of mind. Soc. Dev. 24, 716–733. 10.1111/sode.12116

[B30] Rosen C. (1974). The effects of sociodramatic play on problem-solving behavior among culturally disadvantaged preschool children. Child Dev. 45, 920–927. 10.1111/1467-8624.ep12116929 4143881

[B31] Rubin K. H. Maioni T. L. (1975). Play preference and its relationship to egocentrism, popularity, and classification skills in preschoolers. Merrill Palmer Q. 21, 171–179.

[B32] Saltz E. Dixon D. Johnson J. (1977). Training disadvantaged pre- schoolers on various fantasy activities: effects on cognitive function and impulse control. Child Dev. 48, 367–380. 10.2307/1128629 880835

[B33] Saltz E. Johnson J. (1974). Training for thematic-fantasy play in culturally disadvantaged children: preliminary results. J. Educ. Psychol. 66, 623–630. 10.1037/h0036930

[B34] Schwebel D. Rosen C. Singer J. (1999). Preschoolers' pretend play and theory of mind: the role of jointly constructed pretence. Br. J. Dev. Psychol. 17, 333–348. 10.1348/026151099165320

[B35] Smith P. K. (1988). Children's play and its role in early development: a re-evaluation of the ‘play ethos.’, in Psychological Bases for Early Education, ed Pellegrini A. D. (New York, NY: Wiley), 207–226.

[B36] Smith P. K. Dalgleish M. Herzmark G. (1981). A comparison of the effects of fantasy play tutoring and skills tutoring in nursery classes. Int. J. Behav. Dev. 4, 421–441.

[B37] Suddendorf T. Fletcher-Flinn C. Johnston L. (1999). Pantomime and theory of mind. J. Genet. Psychol. 160, 31–45. 10.1080/00221329909595378

[B38] Taylor M. Carlson S. M. (1997). The relation between individual differences in fantasy and theory of mind. Child Dev. 68, 436–455. 10.2307/1131670 9249959

[B39] Wellman H. M. (2014). Making Minds: How Theory of Mind Develops. New York, NY: Oxford University Press.

[B40] Youngblade L. M. Dunn J. (1995). Individual differences in young children's pretend play with mother and sibling: links to relationships and understanding of other people's feelings and beliefs. Child Dev. 66, 1472–1492. 10.2307/1131658 7555225

